# Systemic Redox Biomarkers in Diabetic Retinopathy: Comparative Evaluation of Thiol–Disulfide Homeostasis, Ischemia-Modified Albumin, and Superoxide Dismutase

**DOI:** 10.3390/diagnostics16213533

**Published:** 2026-10-28

**Authors:** Fatma Sema Akkan Aydogmus, Defne Kalayci, Salim Neselioglu, Goktug Okyar, Ozcan Erel

**Affiliations:** 1 Retina Unit, Department of Ophthalmology, University of Health Sciences Ankara Bilkent City Hospital, Üniversiteler Mahallesi, 1604. Cadde No: 9, Ankara 06800, Türkiye; 2 Department of Medical Biochemistry, University of Health Sciences Ankara Bilkent City Hospital, Ankara 06800, Türkiye; 3 Department of Medical Biochemistry, Ankara Yildirim Beyazit University Faculty of Medicine, Ankara 06800, Türkiye

**Keywords:** diabetic retinopathy, oxidative stress, thiol–disulfide homeostasis, ischemia-modified albumin, superoxide dismutase

## Abstract

**Background/Objectives**: Oxidative stress is implicated in diabetic retinal injury, but systemic redox biomarkers in diabetic retinopathy remain incompletely characterized. This study aimed to evaluate systemic thiol–disulfide homeostasis (TDH), ischemia-modified albumin (IMA), and serum and urinary superoxide dismutase (SOD) activity in patients with diabetic retinopathy (DRP), diabetic individuals without retinopathy, and healthy controls. **Methods**: This cross-sectional comparative study included 31 patients with treatment-naïve DRP, 31 diabetic patients without retinopathy, and 45 healthy controls. Serum native thiol, total thiol, disulfide, IMA, and serum and urinary SOD activities were measured at a single study visit and compared among groups. **Results**: Serum native thiol, total thiol, and disulfide concentrations differed among the three groups (all overall *p* ≤ 0.001). Compared with diabetic patients without retinopathy, patients with DRP had lower native thiol concentrations after Bonferroni correction (*p* = 0.011); the difference in total thiol did not meet the corrected significance threshold (*p* = 0.029). Both diabetic groups had lower disulfide concentrations than healthy controls. IMA concentrations and serum and urinary SOD activities did not differ significantly among groups (all *p* > 0.05). Of the 31 patients with DRP, 15 had diabetic macular edema (DME), whereas 16 did not have DME. No statistically significant differences in the measured redox biomarkers were detected between patients with and without DME. **Conclusions**: Lower serum native thiol concentrations were associated with diabetic retinopathy in this cross-sectional cohort, whereas IMA and serum and urinary SOD activities showed no significant between-group differences. These findings suggest that circulating thiol status warrants further investigation in larger, longitudinal studies.

## 1. Introduction

Diabetes mellitus (DM) is a chronic multisystem metabolic disorder and remains a major contributor to visual impairment worldwide. Diabetic retinopathy (DRP), one of its most frequent microvascular complications, affects a substantial proportion of people living with diabetes and its global burden is expected to increase as diabetes prevalence rises [1,2,3]. Although DRP has traditionally been considered predominantly a microvascular disorder, contemporary concepts increasingly describe diabetic retinal disease as a neurovascular process in which vascular dysfunction, inflammation, neuronal injury, metabolic dysregulation, and oxidative stress interact over time [4,5,6]. This broader view is clinically relevant because molecular abnormalities may precede or accompany the structural and vascular changes that become evident during routine ophthalmic examination.

Oxidative stress is a central component of this pathophysiological network. The retina is particularly susceptible to oxidative injury because of its high metabolic demand and oxygen consumption, abundance of polyunsaturated fatty acids, and continuous requirement for tightly regulated redox signaling [4,5,7,8,9,10]. Persistent hyperglycemia can increase reactive oxygen species (ROS) generation through mitochondrial dysfunction and several interconnected pathways, including the polyol and hexosamine pathways, protein kinase C activation, advanced glycation end-product formation, and lipid peroxidation [4,5,7,8]. Oxidative stress also interacts bidirectionally with inflammatory signaling, microglial activation, endothelial dysfunction, and neurodegeneration, thereby contributing to blood–retinal barrier impairment and progressive neurovascular damage [6,9,10,11,12]. These mechanisms have stimulated interest in biomarkers that might characterize systemic or ocular redox imbalance and complement conventional clinical and imaging assessment [13,14,15,16,17].

Thiol-containing compounds are an important component of endogenous antioxidant defense. Their sulfhydryl groups undergo reversible oxidation to form disulfide bonds, establishing dynamic thiol–disulfide homeostasis (TDH). This reversible system participates in antioxidant protection, redox signaling, regulation of protein and enzymatic function, detoxification, and cellular responses to oxidative injury [18]. The automated assay developed by Erel and Neşelioğlu permits simultaneous quantification of native thiol, total thiol, and dynamic disulfide concentrations and provides a practical approach for characterizing systemic thiol redox status [19]. Disturbances in TDH have subsequently been described in metabolic and inflammatory conditions, including disorders associated with diabetes [18]. However, data specifically addressing TDH in diabetic retinopathy remain limited. Available studies have suggested an association between TDH parameters and DRP or have demonstrated altered thiol–disulfide balance in ocular samples, but findings have not been uniform across biological compartments [20,21].

Ischemia-modified albumin (IMA) represents a different dimension of oxidative biology. Oxidative or ischemic modification of the N-terminal region of albumin reduces its metal-binding capacity, and IMA has therefore been studied as a circulating marker in ischemic and metabolic disorders. The albumin cobalt-binding assay remains a commonly used analytical approach for its measurement [22]. Clinical studies have reported increased IMA in some cohorts with diabetic retinopathy, although its tissue specificity and ability to distinguish retinal disease from systemic metabolic or vascular influences remain uncertain [23]. Superoxide dismutase (SOD), in contrast, is a major enzymatic antioxidant defense that catalyzes the dismutation of superoxide radicals. The experimental and clinical literature supports an important role for SOD-related antioxidant pathways in diabetic retinal injury, but circulating measurements are heterogeneous and may not directly reflect mitochondrial or intracellular antioxidant activity within retinal tissue [7,8,14,15].

Assessing these markers together may therefore be informative because they represent complementary aspects of redox biology: TDH reflects the circulating thiol pool and reversible redox balance, IMA reflects oxidative/ischemic modification of albumin, and SOD activity reflects an enzymatic antioxidant response. At the same time, systemic biomarkers should not be assumed to represent retinal oxidative stress directly, because circulating concentrations may be influenced by whole-body metabolism, renal handling, inflammatory state, nutritional factors, and other comorbidities [13,14,15,16,17]. Against this background, the present study aimed to evaluate serum TDH indices, IMA concentrations, and SOD activity in both serum and urine in patients with treatment-naïve diabetic retinopathy, comparing the findings with diabetic individuals without retinopathy and healthy controls. We additionally explored whether these systemic redox markers differed according to the presence of diabetic macular edema (DME).

## 2. Materials and Methods

This cross-sectional comparative study was conducted by the Ophthalmology and Medical Biochemistry Departments of a tertiary referral hospital. Participants were prospectively recruited between August 2023 and February 2024, and biomarkers were measured at a single study visit. The study was approved by the Institutional Clinical Research Ethics Committee and conducted in accordance with the Declaration of Helsinki, and all participants provided written informed consent.

Participants were assessed for eligibility at the ophthalmology outpatient clinic using predefined inclusion and exclusion criteria. The final cohort comprised 31 patients with treatment-naïve diabetic retinopathy (DRP), 31 patients with diabetes but without retinopathy or other notable ocular disease, and 45 healthy controls. Treatment-naïve DRP was defined as no previous intravitreal injection, retinal laser treatment, or vitreoretinal surgery for DRP. Groups were assigned according to diabetes status and ophthalmic examination.

All participants underwent best-corrected visual acuity assessment (Snellen chart), anterior-segment examination, dilated fundus examination, applanation tonometry, and spectral-domain optical coherence tomography (OCT; Cirrus, Carl Zeiss Meditec, Dublin, CA, USA). Fundus fluorescein angiography was used when the diagnosis was uncertain. One ophthalmologist (FSAA) performed the clinical assessments and classified DRP as mild-to-moderate nonproliferative DRP (NPDR), severe NPDR, or proliferative DRP (PDR), based on retinal findings compared with Early Treatment Diabetic Retinopathy Study (ETDRS) reference photographs. Diabetic macular edema (DME) was defined as central macular thickness >350 µm on OCT together with cystoid macular edema.

Participants were excluded for systemic conditions capable of influencing dynamic TDH—namely chronic inflammatory or rheumatologic diseases, hypertension, cardiovascular disease, hepatic or renal impairment, cerebrovascular disease, thyroid disorders, vitamin deficiencies, and malignancy. Also ineligible were smokers, alcohol users, persons whose body mass index exceeded 30 kg/m^2^, and anyone taking vitamin or antioxidant supplements, oral contraceptives, or hormone replacement therapy.

Once eligible participants had provided signed consent, blood was drawn into standard tubes and left to clot at ambient temperature. Within an hour of collection, serum was obtained by spinning the samples at 1600× *g* for 10 min. The resulting serum was divided into aliquots to prevent repeated freezing and thawing and kept at −80 °C pending analysis.

Urine samples were centrifuged at 4000 rpm to remove cellular debris; supernatants were stored at −80 °C until analysis. Samples with insufficient volume or unsuitable material were excluded from urinary SOD analysis.

A single pyrogallol-based spectrophotometric technique served to quantify SOD activity in both serum and urine. To curtail inter-assay variability, all specimens were processed within one analytical run.

Serum samples were assayed for native thiol, total thiol, disulfide, and IMA concentrations, as well as SOD activity.

Thiol–disulfide homeostasis was assessed using the automated spectrophotometric method of Erel and Neşelioğlu [19]. Disulfide bonds were reduced with sodium borohydride, excess reductant was quenched with formaldehyde, and thiol groups were quantified using DTNB (5,5′-dithiobis-(2-nitrobenzoic acid)). Native and total thiol concentrations were measured, and disulfide was calculated as (total thiol-native thiol)/2. The disulfide/native thiol ratio (SS/SH) was expressed as 100 × disulfide/native thiol.

IMA was detected by means of the albumin cobalt binding assay. Here, 50 µL of 0.1% cobalt (II) chloride (CoCl_2_·6H_2_O; Sigma-Aldrich Chemie GmbH, Riedstrasse 2, Steinheim, Germany) was combined with 200 µL of the patient’s serum. The mixture was gently agitated and then left for 10 min to allow albumin and cobalt to bind. Subsequently, 50 µL of dithiothreitol (DTT) at 1.5 mg/mL was introduced, and the solution was mixed once more. A further 2-min incubation followed. Binding capacity was then lowered by adding 1.0 mL of 0.9% sodium chloride. A parallel control was assembled identically except that distilled water took the place of dithiothreitol. Absorbance was read on a spectrophotometer at 470 nm and reported as absorbance units (ABSU) [22].

SOD activity was measured in serum and urine using a pyrogallol autoxidation inhibition assay based on the Marklund–Marklund method [24]. Stock reagents comprised 0.1 M Tris–cacodylate buffer (pH 8.5), 9.8 mM DETAPAC (adjusted to pH 7.0), and 2 mM pyrogallol in 0.01 N HCl, stored at −20 °C until use. The working reagent combined 8.3 mL of Tris–cacodylate buffer with 1.7 mL of DETAPAC; final reaction concentrations were 50 mM buffer, 1 mM DETAPAC, and 0.2 mM pyrogallol. Reaction volumes were 25 µL of sample, 30 µL of diluent, and 150 µL of reagent. Absorbance was measured at 420 nm (Shimadzu UV-1800, Kyoto, Japan). Activity was inferred from inhibition of pyrogallol autoxidation relative to the uninhibited reaction and reported in U/L, as confirmed by the collaborating laboratory. The reference method defines one unit as 50% inhibition of autoxidation.

Assay reporting and normalization: IMA results are reported as unadjusted albumin cobalt-binding absorbance units (ABSU). Serum and urinary SOD activities are reported in U/L; urinary SOD values were analyzed without creatinine normalization. Serum albumin and urinary creatinine were measured during routine clinical assessment, but their values were not included in the respective adjusted biomarker analyses.

Statistical analysis: Analyses were performed using SPSS version 23.0 (IBM Corp., Chicago, IL, USA). Continuous variables are summarized as mean ± standard deviation (SD), with 95% confidence intervals (CIs) for group means where available; categorical variables are presented as frequencies and percentages. Distributional assumptions were assessed using the Shapiro–Wilk test. The Kruskal–Wallis test was used for overall comparisons of thiol–disulfide parameters, with Mann–Whitney U tests for the three prespecified pairwise group comparisons. Pairwise *p* values are reported as unadjusted values, and a Bonferroni-corrected threshold of *p* < 0.0167 (0.05/3) was used to interpret these contrasts. IMA and SOD group comparisons were evaluated using one-way ANOVA. Categorical variables were compared using the chi-square test. Available-case analyses were used for each variable, without imputation; consequently, analyzable observations could differ by biomarker. Comparisons according to diabetic macular edema (DME) status were exploratory and are interpreted descriptively. Effect sizes are reported for overall tests where they can be calculated from the available output. All tests were two-sided, with *p* < 0.05 used for overall tests. Generative AI (ChatGPT, OpenAI; GPT-6) assisted with English-language editing and preparation of revision text; all content was reviewed by the authors.

## 3. Results

**Figure 1:**
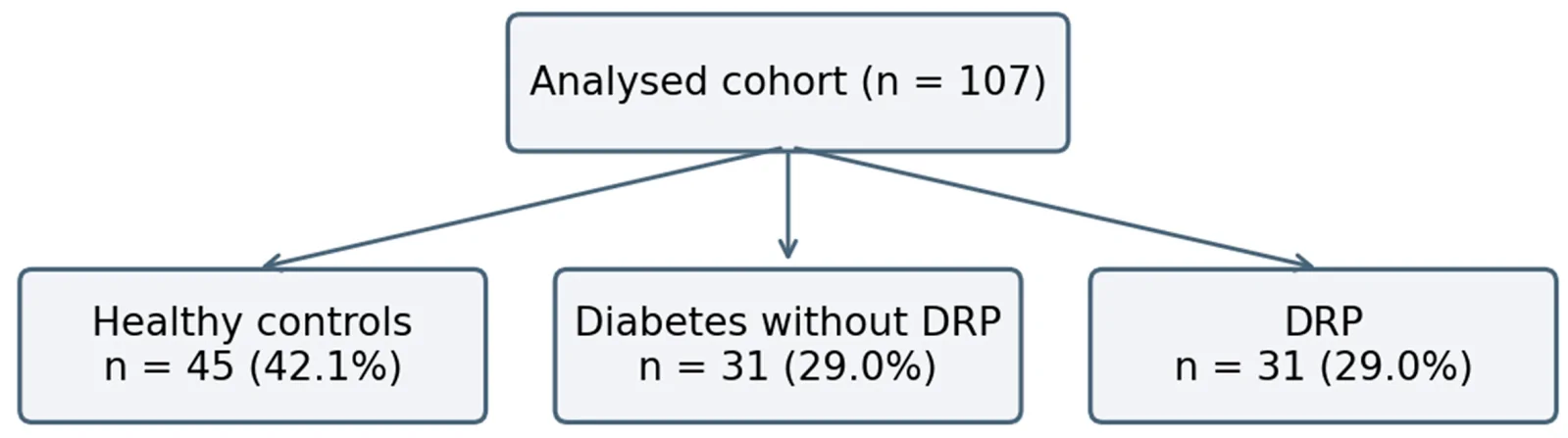
Composition of the analysed cohort (*n* = 107): 45 healthy controls, 31 participants with diabetes without retinopathy, and 31 participants with diabetic retinopathy (DRP). The diagram represents the final analysed cohort.

The analysis encompassed 107 individuals: 31 (29.0%) with treatment-naïve DRP, 31 (29.0%) with DM but no DRP, and 45 (42.0%) healthy controls. The composition of the analysed cohort is shown in Figure 1.

Mean ages came to 60.61 ± 7.1 years (range 45–73) for the DRP group, 61.13 ± 8.1 years (range 44–76) for the DM group, and 58.53 ± 7.8 years (range 50–75) for the controls (*p* = 0.181). Women accounted for 58.1% of the DRP group, 67.7% of the DM group, and 51.1% of controls. Sex distribution did not differ significantly across the groups (*p* = 0.465). Among the 31 patients with DRP, DME was present in 15 patients (48.4%), whereas 16 patients (51.6%) had no DME. Among the 31 patients with DRP, 20 (64.5%) had mild-to-moderate nonproliferative diabetic retinopathy (NPDR), 9 (29.0%) had severe NPDR, and 2 (6.5%) had proliferative diabetic retinopathy (PDR). The severity categories and DME status describe the same 31 participants with DRP; severity-specific biomarker comparisons were not conducted. Baseline demographic characteristics are summarized in Table 1. The expanded baseline table also reports the available retinopathy severity, DME, and prior ocular treatment characteristics for the DRP group.

**Figure 2:**
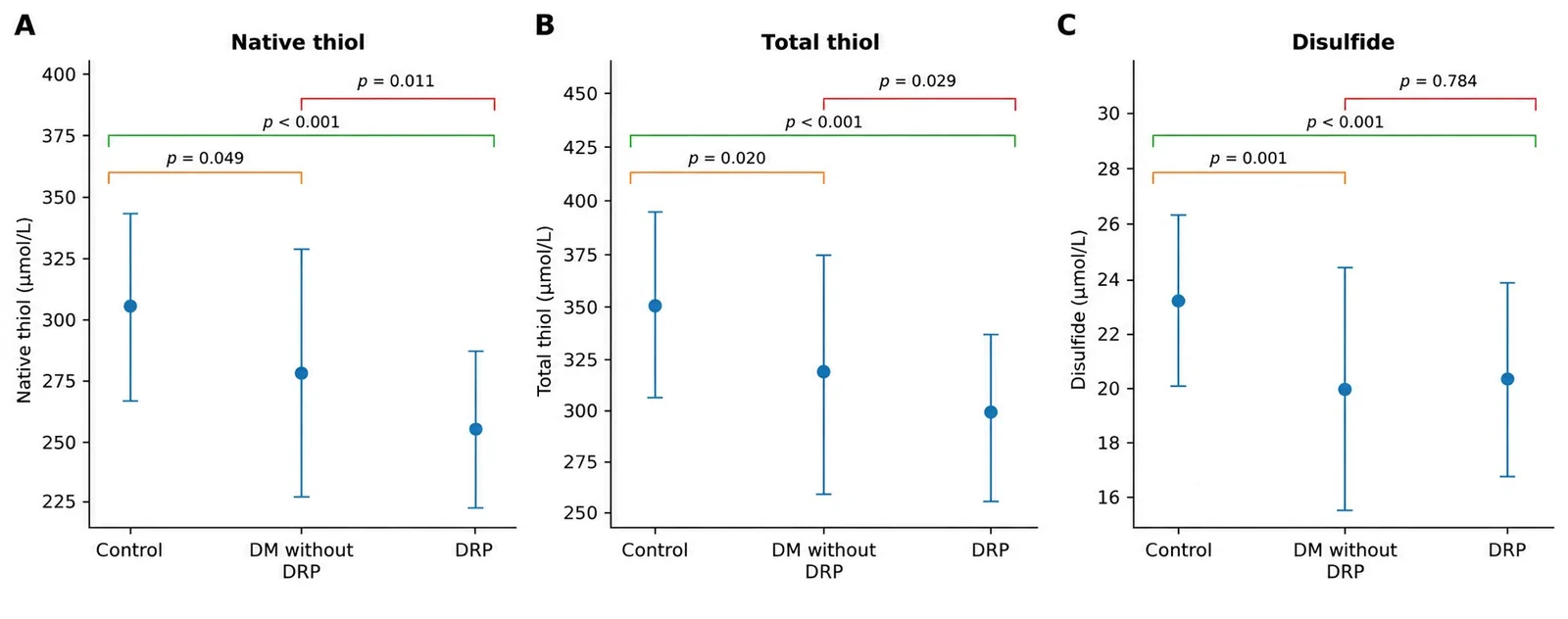
Comparison of serum native thiol (**A**), total thiol (**B**), and disulfide (**C**) concentrations among healthy controls, diabetic patients without diabetic retinopathy (DM), and patients with diabetic retinopathy (DRP). Points represent group means and error bars represent standard deviations. Only pairwise comparisons meeting the Bonferroni-corrected significance threshold (*p* < 0.0167) are indicated as statistically significant.

Serum concentrations of native thiol, total thiol, and disulfide showed significant between-group differences (all *p* ≤ 0.001). In the DRP group, mean native thiol, total thiol, and disulfide concentrations were 258.16 ± 31.05, 298.97 ± 36.35, and 20.40 ± 3.61 µmol/L, respectively; the corresponding figures were 279.72 ± 50.79, 319.76 ± 57.20, and 20.02 ± 4.37 µmol/L in the DM group and 304.16 ± 40.49, 350.68 ± 45.36, and 23.25 ± 3.10 µmol/L in the controls. The corresponding Kruskal–Wallis effect sizes (η^2^H) were 0.197 for native thiol, 0.190 for total thiol, 0.142 for disulfide, and 0.051 for the SS/SH ratio. The biomarker results are summarized in Table 2 and the thiol-related comparisons are illustrated in Figure 2.

Pairwise comparisons were interpreted using a Bonferroni-corrected significance threshold of *p* < 0.0167. Compared with healthy controls, patients with DRP had significantly lower native thiol, total thiol, and disulfide concentrations (all *p* < 0.001). In diabetic patients without DRP, native and total thiol concentrations were numerically lower than those in healthy controls, but these differences did not meet the Bonferroni-corrected significance threshold (*p* = 0.049 and *p* = 0.020, respectively); disulfide concentrations were significantly lower (*p* = 0.001). Native and total thiol concentrations were also numerically lower in patients with DRP than in diabetic patients without retinopathy; after correction for multiple comparisons, the difference remained statistically significant for native thiol (*p* = 0.011), whereas the difference in total thiol did not meet the corrected significance threshold (*p* = 0.029). Disulfide concentrations did not differ between the two diabetic groups (*p* = 0.784).

**Figure 3:**
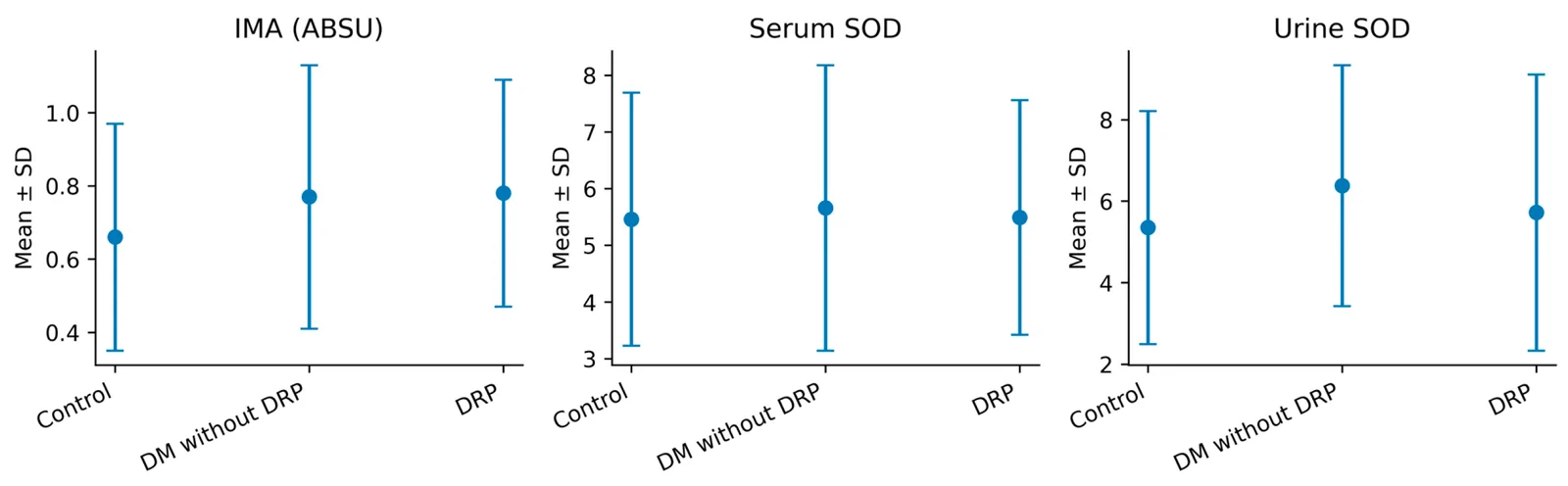
Comparison of ischemia-modified albumin (IMA) concentration, serum superoxide dismutase (SOD) activity, and urinary SOD activity among healthy controls, diabetic patients without diabetic retinopathy (DM), and patients with diabetic retinopathy (DRP). Points represent group means and error bars represent standard deviations. No statistically significant between-group differences were detected for these parameters (all *p* > 0.05). Serum and urinary SOD activities are expressed in U/L.

No statistically significant overall group differences were identified for IMA (ANOVA *p* = 0.200), serum SOD (ANOVA *p* = 0.926), or urinary SOD (ANOVA *p* = 0.408). Exploratory comparisons by diabetic macular edema status showed no statistically significant differences. Table 2 summarizes the laboratory results; Figure 2 depicts the serum thiol comparisons. IMA and SOD comparisons are illustrated in Figure 3.

## 4. Discussion

The present study evaluated systemic TDH, IMA, and serum and urinary SOD activity in patients with treatment-naïve DRP, diabetic individuals without retinopathy, and healthy controls. The principal finding was a graded cross-sectional difference in native and total thiol concentrations across the three groups, with the lowest concentrations observed in patients with DRP. Native and total thiol concentrations were numerically lower in DRP than in diabetes without retinopathy; after correction for multiple comparisons, the pairwise difference remained statistically significant only for native thiol. Disulfide concentrations were reduced in both diabetic groups compared with healthy controls, whereas the disulfide/native thiol ratio showed an overall group difference (*p* = 0.026), without reported post hoc contrasts. In contrast, no statistically significant group differences were detected for IMA concentrations or serum and urinary SOD activity. Taken together, the observed association was more evident for native thiol than for the other measured markers in these unadjusted cross-sectional comparisons.

Oxidative stress provides a biologically plausible framework for these observations. Hyperglycemia-driven ROS generation is closely linked to mitochondrial dysfunction, activation of the polyol and hexosamine pathways, protein kinase C signaling, advanced glycation end-product formation, and lipid peroxidation [4,5,7,8,9,10]. These pathways do not act in isolation. Redox imbalance can amplify inflammatory signaling and microglial activation, while inflammation can in turn promote additional oxidative injury [6,9,10,11,12]. Current models therefore view diabetic retinal disease as a neurovascular disorder in which metabolic stress, inflammation, neuronal dysfunction, and microvascular injury overlap rather than occur as entirely separate events. This framework is relevant to biomarker studies because a circulating marker may capture one component of this network without necessarily tracking all other components or directly mirroring retinal tissue concentrations.

Thiol-containing compounds participate in non-enzymatic antioxidant defenses, but serum thiol concentrations alone do not directly quantify global antioxidant capacity. Dynamic TDH reflects not simply the presence of oxidized molecules but the reversible relationship between reduced thiols and disulfide bonds, a system involved in protein function, signaling, and defense against oxidant exposure [18,19]. In our cohort, native and total thiol concentrations showed a graded cross-sectional pattern, with the highest concentrations in healthy controls, intermediate concentrations in diabetic individuals without retinopathy, and the lowest concentrations in patients with DRP. Although both native and total thiol concentrations were numerically lower in the DRP group than in the DM group, after correction for multiple comparisons the pairwise difference remained statistically significant only for native thiol. The difference between the two diabetic groups is noteworthy because both were exposed to diabetes, whereas clinically detectable retinopathy distinguished the DRP group. This graded pattern supports an association between circulating thiol concentrations and retinopathy status, although its temporal direction remains to be established.

The reduction in disulfide concentrations in both diabetic groups deserves separate interpretation. A simple oxidation model might predict increased disulfide formation when oxidative stress rises. However, sustained oxidative burden may progressively consume the overall thiol pool, leaving less substrate available for reversible disulfide formation. Under such conditions, native thiol, total thiol, and disulfide may decline together rather than demonstrating a straightforward shift toward higher disulfide concentrations. The overall difference in the disulfide/native thiol ratio (*p* = 0.026) does not, without pairwise comparisons, identify which groups differ and does not by itself establish a mechanism. A smaller measurable circulating thiol pool is one possible explanation, but the present measurements cannot distinguish substrate depletion from altered synthesis, turnover, protein concentration, distribution, or other mechanisms. This distinction is important because TDH indices should be interpreted collectively rather than on the basis of disulfide concentration alone [18].

Previous investigations provide an important context for the present serum findings. Coşkun et al. reported an association between TDH parameters and diabetic retinopathy stage [20], whereas Doğruya and Karabağ identified lower total thiol and altered disulfide-related measures in tear fluid from patients with diabetic retinopathy [21]. Our observation that serum native thiol remained different between the two diabetic groups after multiplicity correction extends this discussion to a circulating marker in a treatment-naïve cohort; unlike tear-based measurements, it reflects a systemic rather than ocular-surface compartment. These studies and our results are broadly consistent with altered thiol redox biology in diabetic retinal disease, but differences in the measured compartments preclude direct comparison of absolute biomarker values. Ocular surface measurements may be influenced by local tear-film and ocular-surface processes, whereas circulating thiols reflect systemic metabolism and antioxidant reserve. Differences in specimen type, disease severity, glycemic status, comorbidity exclusions, assay conditions, and sample size may therefore explain why the direction or magnitude of individual TDH parameters varies among studies.

This issue of biological compartmentalization extends beyond TDH. Contemporary reviews of soluble, imaging, ocular, and systemic biomarkers emphasize that diabetic retinopathy is unlikely to be represented adequately by a single circulating analyte [13,14,15,16,17]. Biomarkers measured in serum, urine, tears, aqueous humor, or vitreous may capture different levels of the disease process. Systemic markers are additionally affected by whole-body oxidative metabolism, renal handling, nutrition, medication exposure, and inflammatory or vascular comorbidity. For this reason, the lower circulating thiol concentrations observed here should be interpreted as a circulating biochemical association with DRP, not as a direct quantitative measurement of oxidative stress within retinal tissue. This more conservative interpretation also reduces the risk of overextending a case–control association into a diagnostic or mechanistic claim.

IMA concentrations did not differ significantly among our three groups. This finding contrasts with some reports in which circulating IMA was higher in patients with diabetic retinopathy. Kumar et al., for example, reported higher IMA in patients with DR and a reduction after intravitreal bevacizumab treatment [23]. Several explanations may account for the discrepancy. IMA is not specific to the retina and can be influenced by systemic ischemic, metabolic, vascular, and albumin-related factors. Our study used stringent exclusion criteria for conditions capable of altering systemic redox status, including cardiovascular, renal, hepatic, inflammatory, and other major systemic disorders. Such selection may have reduced non-retinal sources of IMA variation. Differences in DR severity, glycemic control, albumin status, analytical procedures, and statistical power may also contribute. Thus, in contrast to the IMA association reported by Kumar et al. [23], IMA did not show a significant overall group difference in this untreated cohort, while native thiol showed a significant DRP-versus-diabetes contrast after multiplicity correction. This comparison suggests that different systemic redox measures need not provide interchangeable information.

Serum and urinary SOD activities were likewise similar among the groups. SOD is a key enzymatic defense against superoxide radicals, yet its activity in a clinical sample represents the net result of several competing processes. Chronic oxidative exposure may decrease measurable activity through consumption or oxidative inactivation, whereas compensatory responses may increase expression or activity. Moreover, circulating and urinary SOD do not directly represent mitochondrial or intracellular SOD activity in retinal neurons, glia, vascular endothelium, or pericytes. Reviews of oxidative stress in DRP consistently identify impaired antioxidant defense as an important component of disease biology, but individual clinical measurements vary according to specimen, disease stage, assay, and population [7,8,14,15]. The absence of a group difference in serum or urinary SOD in our cohort can therefore coexist with the observed thiol abnormalities because these measurements capture different components of antioxidant physiology.

The broader oxidative-stress literature further illustrates this heterogeneity. Circulating lipid-peroxidation markers such as malondialdehyde have been examined in case–control studies and meta-analyses, while metabolomic studies have identified multiple pathways potentially linked to oxidative stress in DRP [25,26]. These findings reinforce the concept that redox disturbance is multidimensional rather than reducible to a single analyte. From a diagnostic-development perspective, a marker is most useful when it adds information beyond established clinical variables and performs reproducibly populations [25, 26] The present results identify circulating native thiol as a candidate association for validation in longitudinal and externally replicated cohorts, rather than as an established diagnostic marker.

Among patients with DRP, TDH indices, IMA concentrations, and serum and urinary SOD activities did not differ significantly according to the presence of DME. DME itself has a complex pathophysiology involving vascular permeability, inflammation, oxidative stress, and neurodegenerative mechanisms [27]. The absence of subgroup differences in our cohort may indicate that the measured systemic markers are not strongly determined by DME status, but this interpretation must remain cautious because the study was not powered specifically for DME subgroup analysis. A lack of statisticallignificance in a modest subgroup should not be interpreted as proof of biological equivalence [27]. Future studies designed around DME should incorporate adequate sample-size calculations, quantitative OCT parameters, and potentially longitudinal measurements before and after treatment.

The translational relevance of systemic oxidative biomarkers lies in their potential to complement, rather than replace, established ophthalmic assessment. Current DRP evaluation relies on clinical examination and retinal imaging, while research increasingly explores soluble biomarkers, metabolomic signatures, inflammatory mediators, and imaging-derived features for earlier detection and risk stratification [13,14,15,16,17,28,29,30,31]. A clinically useful systemic biomarker would need reproducible analytical performance, clear incremental value over conventional risk factors, and validation across diabetes types, disease stages, and treatment settings. Within that framework, TDH is attractive because it can be measuredith an automated assay and reflects an integrated thiol redox system [18,19,19,28,29,30,31]. However, the present study should be considered hypothesis-generating; it does not establish TDH as a stand-alone diagnostic test.

An important contribution of this study is its parallel assessment of TDH, IMA, and SOD activity in serum and urine across treatment-naïve DRP, diabetes without retinopathy, and healthy controls. This within-cohort comparison places the native-thiol association alongside non-significant overall IMA and SOD findings under the same clinical grouping, rather than interpreting each biomarker in isolation. Together with the contrast between serum-based findings here and tear-based TDH observations [21], the results underscore the importance of biological compartment and marker selection when designing future DRP biomarker studies.

The study also has implications for future design. Longitudinal cohorts could determine whether reduced native or total thiol precedes clinically detectable DRP, tracks progression across standardized severity levels, or changes after systemic metabolic improvement or ocular therapy. Simultaneous sampling of systemic and ocular compartments would help clarify how circulating TDH relates to local retinal redox biology. Integration with OCT and OCT angiography, glycemic indices, renal function, lipid profiles, and inflammatory biomarkers could determine whether thiol measures contribute independent information. Repeated measurements would also help distinguish stable inter-individual differences from dynamic changes associated with disease activity. Such work would move the field from cross-sectional association toward clinically interpretable biomarker validation.

The treatment-naïve status of the DRP group is also relevant when interpreting the biomarker profile. Intravitreal anti-VEGF agents, corticosteroids, laser treatment, and other ocular interventions can alter local inflammatory or vascular activity and may indirectly complicate comparison of systemic biomarkers. By evaluating patients before ocular treatment, the present study reduces one source of treatment-related heterogeneity. The observed thiol differences were therefore documented in association with untreated retinal disease. Future studies should record these systemic variables prospectively and examine whether TDH remains independently associated with DRP after multivariable adjustment. Repeated measurements before and after treatment could also help determine whether systemic thiol indices are stable patient characteristics or dynamic markers that change with disease activity or therapeutic response.

Our stringent exclusion criteria represent both a methodological strength and a consideration for external validity. Excluding smoking, obesity above the prespecified threshold, antioxidant supplementation, and systemic disorders known to influence redox biology was intended to reduce non-retinal sources of variation in TDH, IMA, and SOD. Such restriction can improve internal interpretability in a mechanistic biomarker study, but it also creates a cohort that may not fully represent the broader population with diabetes, in whom hypertension, cardiovascular disease, renal dysfunction, obesity, and polypharmacy are common. Consequently, the magnitude of between-group differences observed here should not be assumed to generalize directly to unselected clinical populations. Replication in larger real-world cohorts, followed by analyses that adjust for common comorbidities rather than excluding them, will be important before considering systemic thiol indices for clinical risk stratification.

Strengths of this work include prospective recruitment, inclusion of both diabetic and healthy comparison groups, enrollment of treatment-naïve DRP patients, concurrent assessment of TDH, IMA, and serum and urinary SOD activity, and stringent exclusion of systemic conditions capable of substantially influencing redox status. The use of the same analytical run for SOD measurements and standardized preanalytical handling were additional measures intended to limit laboratory variability. Evaluating markers representing different redox domains also allowed us to observe that thiol indices differed among groups even when IMA and SOD did not.

Interpretation of these indices requires care because disulfide is mathematically derived from native and total thiol rather than independently measured. Concurrent reductions in all three absolute concentrations, with an overall difference in the disulfide/native thiol ratio but no reported pairwise contrasts, are compatible with contraction of the overall measurable thiol pool; they do not establish the biochemical mechanism responsible.

Study Limitations: This cross-sectional study included modest DRP and DME subgroups and cannot establish temporal or causal relationships. Incomplete data on diabetes duration, glycemic control, renal function, albumin, and medication use limited adjustment for confounding. IMA was not albumin-adjusted, and urinary SOD was not normalized to creatinine. Fasting status, collection time, hemolysis screening, laboratory blinding, and assay order were not documented. Study-specific assay validation and original SOD calculation records were unavailable. Consecutive enrollment and a shared source population could not be confirmed. Urinary SOD denominators also require reconciliation with the final cohort. These factors may affect measurement comparability and generalizability.

## 5. Conclusions

In this cross-sectional study, serum native thiol concentrations were lower in patients with diabetic retinopathy than in diabetic participants without retinopathy after correction for multiple comparisons. IMA and serum and urinary SOD activities showed no significant overall between-group differences. Larger longitudinal studies with validated analytical data and adjustment for relevant confounders are needed to assess the reproducibility and clinical relevance of these findings.

## Figures and Tables

**Table 1:** Baseline demographic and documented clinical characteristics of the study groups. Values are mean ± standard deviation, range, or percentage as indicated. The control-group female proportion is calculated using all 45 participants (23/45). Retinopathy severity, DME status, and prior ocular treatment status apply to the DRP group only.

Characteristic	Control (*n* = 45)	DM Without DRP (*n* = 31)	DRP (*n* = 31)	*p*
Age, years	58.53 ± 7.8	61.13 ± 8.1	60.61 ± 7.1	0.181
Age range, years	50–75	44–76	45–73	—
Women, *n*/N (%)	23/45 (51.1)	21/31 (67.7)	18/31 (58.1)	0.465
Diabetes status	No diabetes	Diabetes without DRP	Diabetes with DRP	—
DRP: mild-to-moderate NPDR, *n* (%)	Not applicable	Not applicable	20/31 (64.5)	—
DRP: severe NPDR, *n* (%)	Not applicable	Not applicable	9/31 (29.0)	—
DRP: PDR, *n* (%)	Not applicable	Not applicable	2/31 (6.5)	—
DME present, *n* (%)	Not applicable	Not applicable	15/31 (48.4)	—
DME absent, *n* (%)	Not applicable	Not applicable	16/31 (51.6)	—
Prior ocular DRP treatment	Not applicable	Not applicable	None (31/31 treatment-naïve)	—

**Table 2:** Comparison of thiol–disulfide and ischemia-modified albumin concentrations and superoxide dismutase activity among the study groups. Values are mean ± SD; 95% CIs for group means are shown where available. Pairwise *p* values for thiol parameters are unadjusted Mann–Whitney U *p* values (Control vs. DM/Control vs. DRP/DM vs. DRP); the Bonferroni-corrected threshold is *p* < 0.0167. Overall thiol comparisons use Kruskal–Wallis tests, and IMA and SOD comparisons use one-way ANOVA. η^2^H and η^2^ denote the corresponding effect sizes. IMA is reported in ABSU and SOD activity in U/L.

Parameter	Control (n = 45)	DM Without DRP (n = 31)	DRP (n = 31)	Overall *p*/Effect Size	Pairwise *p* ^a/b/c^
Native thiol (µmol/L)	304.16 ± 40.49 (95% CI 292.00–316.33)	279.72 ± 50.79 (95% CI 261.09–298.34)	258.16 ± 31.05 (95% CI 246.78–269.55)	<0.001/η^2^H = 0.197	0.049/<0.001/0.011
Total thiol (µmol/L)	350.68 ± 45.36 (95% CI 337.06–364.31)	319.76 ± 57.20 (95% CI 298.78–340.74)	298.97 ± 36.35 (95% CI 285.64–312.31)	<0.001/η^2^H = 0.190	0.020/<0.001/0.029
Disulfide (µmol/L)	23.25 ± 3.10 (95% CI 22.33–24.19)	20.02 ± 4.37 (95% CI 18.42–21.62)	20.40 ± 3.61 (95% CI 19.08–21.73)	<0.001/η^2^H = 0.142	0.001/<0.001/0.784
Disulfide/native thiol (%)	7.67 ± 0.76 (95% CI 7.45–7.90)	7.30 ± 1.58 (95% CI 6.72–7.88)	7.90 ± 1.09 (95% CI 7.50–8.30)	0.026/η^2^H = 0.051	—
IMA (ABSU)	0.66 ± 0.31 (95% CI 0.57–0.75)	0.77 ± 0.36 (95% CI 0.64–0.90)	0.78 ± 0.31 (95% CI 0.67–0.90)	0.200/η^2^ = 0.030	—
Serum SOD activity (U/L)	5.46 ± 2.23 (95% CI 4.79–6.14)	5.66 ± 2.52 (95% CI 4.74–6.59)	5.49 ± 2.07 (95% CI 4.74–6.25)	0.926/η^2^ = 0.001	—
Urinary SOD activity (U/L)	5.35 ± 2.86	6.38 ± 2.96	5.72 ± 3.39	0.408	—

^a^ Significance between Control and Dry AMD group (pairwise comparison). ^b^ Significance between Control and Neovascular AMD group (pairwise comparison). ^c^ Significance between Dry AMD and Neovascular AMD group (pairwise comparison).Data are presented as mean ± standard deviation (range).

## Data Availability

The data presented in this study are available on request from the corresponding author due to privacy and ethical restrictions.
